# Preventing obesity-related cancer with the revolution in obesity management: the challenges of undertaking a clinical trial and potential solutions

**DOI:** 10.1038/s41416-026-03355-8

**Published:** 2026-02-24

**Authors:** Matthew Harris, Julia Brown, Andrew G. Renehan

**Affiliations:** 1 Division of Cancer Sciences, School of Medical Sciences, Faculty of Biology, Medicine and Health, University of Manchester, Manchester, UK; 2 Leeds CRUK Clinical Trials Unit, Leeds Institute of Clinical Trials Research, University of Leeds, Leeds, UK; 3 Manchester Cancer Research Centre, NIHR Manchester Biomedical Research Centre, Manchester, UK; 4 Colorectal and Peritoneal Oncology Centre, Christie NHS Foundation Trust, Manchester, UK

**Keywords:** Cancer prevention, Obesity, Clinical trial design

## Abstract

Evidence from conventional and Mendelian Randomisation epidemiological studies support the conclusion that obesity is causally associated with increased risk of several common cancer types. Some evidence, notably from quasi-experimental bariatric surgery studies, support the concept that sustained long-term weight loss in individuals is associated with reduction of cancer incidence, particularly in women. Yet, there are no authoritative public health policies directed specifically at large-scale weight management interventions to prevent obesity-related cancers. At least two adversities conspire against public health success: (i) awareness of the causal link between obesity and cancer risk; and (ii) lifestyle interventions are associated with only moderate weight loss that is generally not sustained long enough to result in clinically meaningful cancer prevention. However, there is now a revolution of effective pharmacotherapy for obesity, namely glucagon-like-peptide (GLP)-1 agonists and their extended family of dual and triple agonists, which leads to substantial rates of weight loss, sustained while individuals continue to take the drug. There is now a key new cancer prevention research question, whether this drug class might significantly reduce cancer risk with long-term use. The logistics of addressing this question in a clinical trial setting are discussed and potential strategies to overcome these challenges are proposed.

## Introduction

Obesity is one of the most pressing global health challenges of the 21st century, with its prevalence rising at an alarming rate. In 2016, more than 1.9 billion adults were classified as overweight, including over 650 million individuals living with obesity, a number projected to surpass 1 billion by 2030 [1]. Despite decades of public health efforts, obesity rates continue to climb in nearly every country. Between 1990 and 2022, 89% (177) of all countries had increasing prevalence of obesity in women, and 73% (145) had increasing prevalence in men [2]. This rise is fuelling a parallel increase in obesity-related diseases, including type 2 diabetes [3], cardiovascular disease [4], and, critically, cancer.

In 2016, the International Agency for Research on Cancer published a consensus on the strength of evidence for cancer types that are directly affected by excess body adiposity. They defined 13 individual obesity-related cancers (oesophageal adenocarcinoma, gastric cardia, colorectal, liver, gallbladder, pancreas, post-menopausal breast, corpus uteri, ovarian, renal-cell, meningioma, thyroid and multiple myeloma) [5]. Causal pathways are yet to be fully understood but are considered likely to be multifactorial, including: immune modulation, chronic inflammation, insulin resistance and hormonal imbalance.

Consistent with a casual link between obesity and cancer risk, in parallel with rising global prevalences of excess body adiposity, rates of cancer and obesity-related cancer are rising. In an analysis of the GLOBOCAN database; in the UK, crude cancer rates have steadily risen between 2003 and 2017 (2003: males =  574.8 per 100,000, females = 514.9 per 100,000; 2017: males = 689.3 per 100,000, females = 598.4 per 100,000). When selected for obesity-related cancers (2003: males = 164.3 per 100,000, females = 318.5; 2017: males = 196.7 per 100,000, females = 358.0), this steady increase is again demonstrated[6].

**Fig. 1:**
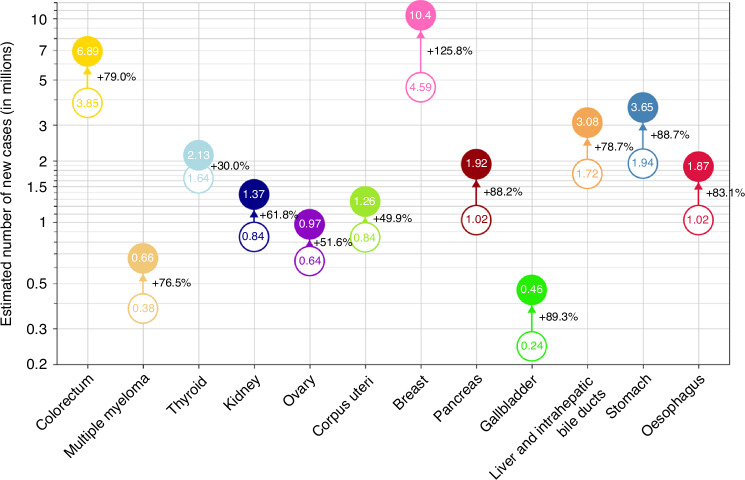
Predicted annual incidence of obesity-related cancer between 2022 and 2050 from CANCER TOMORROW.

The future of obesity-related cancer is illustrated in GloboCan’s CANCER TOMORROW model, predicting the incidence of site-specific cancers based on forecasted population changes up to 2050. Even if obesity rates remain static from today, the number of annual new cases of obesity-related cancer will considerably increase over the next 25 years across the world, further illustrating the potential future healthcare burden (Fig. 1) [7].

### Reversing obesity-related cancer risk

Current policy and evidence reviews indicate that overweight and obesity are causally associated with cancer and that excess body weight should be avoided. However, there is a lack of understanding whether this risk is reversible in those living with obesity; does losing this weight reduce obesity-related cancer risk? It stands to reason that treating obesity might reduce an individual’s risk of obesity-related cancer, and there are three broad categories of intentional weight loss strategies that could achieve this: bariatric surgery, behavioural intervention (including diet and exercise), and pharmacotherapy. Whilst there have been suggestions from the observational literature that intentional weight loss can reduce cancer risk [8], these studies are limited due to narrowly defined populations and risks of bias.

#### Bariatric surgery

Surgical obesity management produces long-term weight loss in the order of around 25–35% of total body weight [9]. It has also been shown to have a likely protective effect on cancer development that has been demonstrated in meta-analysis of observational studies [10]. However, surgery is unlikely to be a feasible population-level solution, due to the nature of complications, limited healthcare capacity and acceptability.

#### Behavioural intervention

Behavioural intervention, including diet, exercise and psychotherapy have been a mainstay of the treatment of obesity. In comparison to surgery, its weight loss efficacy is modest, in the order of 2–5% of total body weight [11]. So far, there has been no randomised controlled trial evidence (RCT) that has shown a significantly reduced risk of cancer from a behavioural intervention [12]. However, in the LookAHEAD study, an RCT comparing intensive lifestyle intervention (ILI) compared to best medical advice suggested a possible preventative effect on obesity-related cancers after 11 years, but this did not reach statistical significance (RR = 0.84, 95% CI = 0.68–1.04) [13]. The Diabetes Prevention Programme, a large RCT comparing Metformin, ILI and placebo, indicated that after 21 years of follow-up, there was no significant reduction in cancer incidence comparing ILI to placebo (RR 0.96, 95% CI = 0.79–1.18) [14]. Further supporting that if there were a cancer-protective effect of behavioural intervention, it would be very small.

#### Pharmacotherapy

Obesity pharmacotherapy has recently experienced a revolution with the advent of glucagon-like-peptide (GLP)-1 agonists (for example, semaglutide), and subsequently multi-incretin receptor agonists including dual agonists (for example, gastric inhibitory polypeptide (GIP) analogue and a GLP-1 receptor agonist, Tirzepatide) and triple agonists (for example, GLP-1, GIP, and glucagon receptor (GCGR), Retatrutide).

Initially indicated for the management of type 2 diabetes, significant weight-loss was seen. This drug class was therefore tested as a weight losing intervention in individuals with overweight and obesity without type 2 diabetes. Note that in some instances, doses higher than those used in the treatment of type 2 diabetes were chosen (for example, semaglutide 2.4 mg weekly injections). A large set for trials, mainly published in the past 3 years, have consistently demonstrated that this drug class achieves high levels of weight loss over a the period of duration for which the drugs are taken. Trials are currently ~1 to 4.5 years in duration. Weight loss ranges from 12% to 18% in a stepwise manner from single agent GLP-1 agonist to triple agonists therapy (Table 1).

**Table 1:** Estimations of weight loss across the classes of GLP-1 agonist family drugs.

Intervention class	Example	Evidence source	Estimated weight loss
New pharmacotherapy options			
GLP-1 agonist (single)	Semaglutide	Meta-analysis of phase 3 RCTs [14]	~15%
Dual agonist (GLP-1, GIP)	Tirzepatide	RCT, phase 3 extension [15]	~25%
Dual agonist (GLP-1, DACR)	Cagrilintide/semaglutide	RCT, phase 3 [16]	~20%
Triple agonist (GLP-1, GIP, GCGR)	Retatrutide (LY-3437943)	RCT level, phase 2 [17]	~24%
Surgery			
Bariatric surgery	Roux-en-Y gastric bypass	Meta-analysis of long-term observational studies [18]	~22%

*RCT* Randomised controlled trial, *GLP-1* glucagon-like peptide agonist, *GIP* gastric inhibitory polypeptide analogue, *DACR* dual amylin and calcitonin receptor agonist, *GCGR* Glucagon receptor agonist.

RCTs demonstrate their benefits beyond weight loss, on reducing the risk of obesity-related co-morbidities such as type 2 diabetes [15], cardiovascular events [16], renal function [17], and osteoarthritis [18]. Their safety in relation to cancer has been demonstrated in series of meta-analyses of RCTs [19], and early concerns of risks of thyroid and pancreatic cancer have been reassured. GLP-1 receptor agonists may now be considered as an obesity-related cancer prevention method, which could be critical in the future of obesity and cancer management.

A matched cohort analysis of GLP-1 use and cancer risk showed significant reductions in the risk of several cancers [20]. However, there are a number of methodological concerns of underlying bias, demonstrating the challenges in eliciting the effect of cancer chemo-prevention with observational techniques. Currently, there is a lack of evidence with the strength to inform clinical practice and public health policy.

### The need for a clinical trial

Given the difficulty of undertaking observational research and its feasibility to have the strength to influence practice change, we must look to other methods to understand this gap. RCTs have previously been successful in influencing management and public health policy, with examples in the Diabetes Prevention Programme [21], DiRECT [22] and DROPLET [23] trials. Impressive findings from the STEP [24], SURMOUNT [25] and SELECT [16] trials have led to the licencing and widespread use of GLP-1/dual receptor agonists for the treatment of obesity. An RCT assessing the effect of these drugs on cancer risk could be influential and vital in implementing evidence-based cancer prevention strategies.

### Clinical trial challenges

A clinical trial investigating GLP-1 receptor agonist effects on cancer risk will be complex and challenging for a variety of reasons which are explored in Table 2. The National Cancer Institute USA explored the feasibility of a weight loss intervention for breast cancer prevention clinical trial [26], and an endpoint of survivorship, rather than incidence was deemed most feasible and was pursued [26, 27].

**Table 2:** Broad clinical trial design challenges.

Trial design category	Challenges
Population	1. Selecting a generic population will maximise external validity but may lead to a low absolute risk of developing obesity-related cancer 2. High cancer risk populations may have other comorbidity, that may be modified by the tested intervention leading to longer survival and potentially greater chance of developing an incident cancer
Intervention	1. GLP-1 receptor agonists currently have no long-term data for side effects and tolerability beyond 4 years 2. Drug intervention may have to be given with a parallel behavioural intervention to ensure maximal long term weight loss, adding to the complexity of a trial 3. A drug intervention delivery may come with associated high financial costs due to close monitoring of side effects and non-cancer outcomes for the duration of a trial
Comparison	1. A pure placebo-controlled arm may not be acceptable to patients, given well documented health benefits of GLP-1 receptor agonists 2. There may have to be a behavioural intervention delivered to the control group, to ensure acceptability of randomisation, leading to a reduced differential weight loss between intervention and control 3. New and easily accessible alternative weight loss drugs may come onto the market during the trial, leading to contamination
Outcome	1. Obesity related cancer risk in the general population is low, even in higher BMI categories, therefore a detectable relative effect may require large sample sizes 2. Cancer develops over a long period of time, therefore it is theorised that an effect of weight loss on cancer prevention may only take place after a long duration, in the order of 10 or more years

Given these clinical trial design challenges, we explore specific issues and potential solutions in planning a large-scale GLP-1 receptor agonist and cancer prevention trial. We acknowledge that the intervention is complex, likely to require at least 10 years of intervention for the endpoint of cancer incidence, and would include behavioural strategies to supplement the chemoprevention during weight loss intervention and maintenance.

#### Competing risks (Population)

A clinical trial that runs for over a decade may create the possibility of interference from competing risks. The SELECT trial [16] has already demonstrated a probable reduction in cardiovascular mortality of 15% over 4 years. If a participant lives longer, they have a greater chance of developing cancer, closing the gap between intervention and control and masking a potential protective effect on cancer [28]. This also raises the important consideration of whether it is ethical to compare an intervention with a placebo, in circumstances where the intervention has such broadly proven alternative long-term health benefits. It may not be acceptable to participants and researchers to run a 2-arm trial comparing, for example, semaglutide versus placebo for 10 years.

#### Rarity of cancer events (Population)

In a general UK/EU population, estimating the baseline cancer risk for a population with a mean BMI > 30 is a challenge, but may be in the order of around 7–8% over 10 years [29]. This rarity will mean that large sample sizes will be required to detect relative differences and avoid error. Taking the most basic example of a 2-arm trial of GLP-1 vs placebo, if the intervention reduced the risk of all cancers by an optimistic 25%, it would still require over 7000 participants in each arm under perfect follow-up and adherence, to give 90% statistical power to detect the small absolute reduction of 2% between intervention and control, equating to a number needed to treat of 50. A long-term trial like this will likely include considerable uncertainties and factors that will mean a basic sample size calculation such as this may be a considerable underestimation.

#### Complexity of intervention (Intervention)

Any pharmacological treatment is considered a complex intervention [30]. This is particularly true with obesity management drugs. They require gradual escalation of dosing, well-documented side effects and when stopped, participants tend to put their weight back on. In the STEP trials [24], the medications are given alongside a behavioural intervention, creating further complexity in the standardisation and delivery of the intervention as a whole. It must also be considered whether these medications are delivered consistently over the entire duration of a trial, or whether they are given to achieve a certain weight loss, and then a maintenance intervention is used to aim to keep this weight off. These decisions will be influential in intervention and control compliance, adherence and maintenance of weight loss, and therefore the overall effect size difference across the duration of a trial.

#### Contamination due to alternative weight loss intervention (Intervention and control)

Since semaglutide was first licenced for the treatment of obesity in 2021, tirzepatide, a GLP-1/GIP receptor agonist with has even greater efficacy of weight loss, has achieved FDA approval in 2023. There are numerous other obesity-management drugs advanced in their pipeline of development that are likely to come into use [31, 32]. Undertaking a clinical trial will require individuals to take a single pre-specified drug and not deviate over the course of follow-up. It is possible that due to the long duration of a proposed trial and likely availability of new medications, that participants may decide to switch, especially if they are experiencing side effects. This is even more likely in participants who are not achieving a satisfactory magnitude of weight loss. This may be more prevalent in the control group, leading to a reduced differential of weight loss between the control and intervention arm. This threat of contamination between weight loss intervention may lead to bias and a considerable loss of statistical power, driving sample size requirements higher and greater chance of trial failure. Furthermore, if strict safety monitoring protocols are required, this may add substantially to the cost and logistical challenges of a clinical trial.

#### Choice of control (Control)

A pure 2-arm, placebo-controlled, RCT between a single drug and placebo may not be acceptable for prospective participants to be randomised into, given documented multi-systemic benefits of GLP-1 administration. Selection of alternative drugs, or behavioural intervention, as a control may lead to significant weight loss and potentially obesity-related cancer-preventative effects thus reducing effect sizes and contributing to reduced statistical power.

#### Long duration of trial needed (Outcome)

Any clinical trial investigating an effect on the change in cancer risk will logically have to take place over a time period in which the carcinogenic environment has been modified for a sufficient amount of time. The sojourn time for many common obesity-related cancer types exceeds 2 years [33–35]. In the SOS study [36], following bariatric surgery, the divergence in cancer risk between groups occurred after around 10 years. It should be expected that a clinical trial of this nature would plan for at least 10 years of follow-up. A potential trial will require large sample sizes and a lengthy duration. This will compound the importance of rates of recruitment [37], adherence [38] and attrition [39].

#### Financial cost (Outcome)

Having established that such a trial will need a lengthy duration of follow-up, large sample sizes, the delivery of a complex intervention and therefore a relatively large burden of support; the financial cost could be very large. A typical phase 3 RCT may require a number of participant visits to a clinic, possibly adding to drop-out rates and recruitment difficulties, compounded by the long-term nature of a cancer prevention trial. The financial and opportunity cost must be considered to assess whether it is in the best interests of the research community, considering associated risks of trial failure.

#### Risk of an infeasible or underpowered trial

Given the aforementioned logistical, ethical and financial issues, a 2-arm RCT comparing the use of GLP-1 drugs for cancer prevention may not be feasible in the real world. However, there are several potential solutions to mitigate these issues that are outlined in Table 3. Despite factoring in novel solutions, there will remain a risk of an underpowered trial, not reaching a firm conclusion, that could be damaging to research progression for the reduction of obesity-related cancer incidence.

**Table 3:** Clinical trial design challenges and potential solutions.

Challenge	Potential solution	Solution challenges
Competing risks	*Competing risk analysis*: Prespecified competing risks could be accounted for in a competing risk analysis [28, 40].	Competing risks are not necessarily identifiable up front, prior to a clinical trial and pre-specified competing risk analyses may not fully capture the overall non-cancer health benefit of a drug within a clinical trial setting such as this.
Rarity of cancer events	*Selective population*: selecting a population with a higher baseline risk of obesity-related cancer raises the potential for detection of a significant effect size at smaller sample sizes. This may be a population such as category 3 obesity in women or participants with a genetic high risk (e.g. BRCA)	A super-selected population may have a higher baseline risk however this may create a lack of external validity for extrapolation to a general population [41]. Those at a genetic predisposition may not have a risk that is modifiable by the treatment of obesity.
Complexity of intervention	*Novel trial designs*: Factorial [42], multiphase optimisation strategy trials [43] (MOST) or adaptive intervention [44] designs may allow for refinement of the intervention and assessment of component parts over time. This may be beneficial if the intervention requires the administration of a drug and behavioural intervention at the same time. *PPIE involvement*: Having prospective trial participants involved in the design of intervention may allow for an optimisation and improvements in acceptability, subsequently having positive impacts of attrition and adherence rates.	The utilisation of novel trial designs and inclusion of increasingly complex intervention options may add to the financial burden of the trial. Specific expertise will be required, adding to training burden and potentially to the trial participants [45]. Complex designs including multiple arms, as in factorial or SMART trials may add to dilution of the power of a trial.
Contamination due to alternative weight loss intervention	*Platform trial* [46]: A multi-arm trial such as a platform design, will allow for flexibility of new drugs or treatments being developed, especially if efficacy is greater (e.g tirzepatide compared to semaglutide).	The addition of new interventions on a stable recruitment rate will reduce the potential power of detecting a true effect if compared to an inactive or placebo group. Adding complexity to the trial may increase costs and the overall duration.
Choice of control	*PPIE involvement* [47]: The involvement of patients and prospective participants in the clinical trial design decisions will allow for the best chance of optimising these important aspects, including the nature of follow up, frequency of clinic visits. Strong PPIE inclusion will be vital in designing a successful long term clinical trial.	With no prior clear evidence of expected recruitment, adherence and attrition rates of GLP-1 drugs long term, the magnitude and consistency of weight loss could differ from what is expected. These differences could affect the chance of a type 2 error despite prior discussion and intervention design with patients and public. There are relatively few established patient organisations for people living with obesity globally and may create challenges recruiting in the international setting.
Long duration needed	*Surrogate endpoint* [48]**:** The identification and selection of a cancer pre-cursor or surrogate endpoint for cancer incidence may mitigate the need for the long duration of a clinical trial, on the principle that if pre-cursors are reduced, so will the likelihood of an incident cancer.	Extrapolation of the cancer pre-cursor or biomarker to an incident cancer may not necessarily have direct correlation in a real world situation [49]. Individual cancers may have different pre-cursor endpoints. Currently there is an absence of useable generalised pre-cursors or biomarkers that could be used in this context.
High financial cost	*Multiple endpoints* [50]: The design of a clinical trial assessing non-cancer outcomes may add to the efficiency and overall benefit to the research field.	Inclusion of non-cancer outcomes will add to the complexity of a clinical trial, diverting resources away from the cancer outcome of interest. It will also make power calculations difficult and could ultimately affect the chance of a type 2 error.
Lack of feasibility	*TARGET trial emulation* [51]: Designing an RCT that aims to answer whether pharmacotherapy may reduce obesity-related cancer risk may prove to be infeasible. However, simulations such as in a TARGET trial framework may provide a way forward	A TARGET observation study may still face the same methodological issues as previous observational studies.

## Conclusion

In the next 25 years, the rising rates and burden of obesity and obesity-related cancer on healthcare systems across the world will be considerable. Cancer prevention methods should be considered, and a new category of pharmaceutical weight loss provides a potentially promising potential chemo-prevention strategy. As it stands, evidence to inform clinical management and public health policy is lacking and observational studies have been proven to be challenging to undertake and extrapolate into this arena. A clinical trial could be definitive and influence the future of obesity-related cancer prevention, but it will be lengthy, complex and costly. Despite currently high drug costs, cancer prevention offers a likely dominant cost-effective result due to the expense, complexity and long-term nature of cancer treatment.

We have highlighted key methodological challenges with undertaking an obesity-related cancer chemoprevention trial alongside potential solutions. A thorough feasibility analysis in parallel with research into mechanistic pathways and potential bio-marker and cancer pre-cursors should be undertaken to progress this field. An evidence-based pharmacological obesity-related cancer prevention strategy could be widely impactful on the future burden to healthcare systems across the world.
